# A Structured Pathway Toward Disruption: A Novel HealthTec Innovation Design Curriculum With Entrepreneurship in Mind

**DOI:** 10.3389/fpubh.2021.715768

**Published:** 2021-09-03

**Authors:** Holger Fritzsche, Beatrice Barbazzeni, Mohd Mahmeen, Sultan Haider, Michael Friebe

**Affiliations:** ^1^INKA (Intelligente Katheter) - HealthTec Innovation Laboratory, Medical Faculty, Otto-Von-Guericke-University, Magdeburg, Germany; ^2^European Structural and Investment Funds-International Graduate School (ESF-GS) Analysis, Imaging, and Modelling of Neuronal and Inflammatory Processes (ABINEP) International Graduate School, Otto-Von-Guericke-University, Magdeburg, Germany; ^3^Siemens Healthineers, Erlangen, Germany

**Keywords:** biodesign, design thinking, health democratization, bioengineering education, disruptive technologies, exponential medicine, future of health, twenty-first century skills

## Abstract

The typical curriculum of training and educating future clinicians, biomedical engineers, health IT, and artificial intelligence experts lacks needed twenty first-century skills like problem-solving, stakeholder empathy, curiosity stimulation, entrepreneurship, and health economics, which are essential generators and are pre-requirements for creating intentional disruptive innovations. Moreover, the translation from research to a valuable and affordable product/process innovation is not formalized by the current teachings that focus on short-term rather than long-term developments, leading to inaccurate and incremental forecasting on the future of healthcare and longevity. The Stanford Biodesign approach of unmet clinical need detection would be an excellent starting methodology for health-related innovation work, although unfortunately not widely taught yet. We have developed a novel lecture titled HealthTec Innovation Design (HTID) offered in an interdisciplinary setup to medical students and biomedical engineers. It teaches a future-oriented view and the application and effects of exponential trends. We implemented a novel approach using the Purpose Launchpad meta-methodology combined with other innovation generation tools to define, experiment, and validate existing project ideas. As part of the process of defining the novel curriculum, we used experimentation methods, like a global science fiction event to create a comic book with Future Health stories and an Innovation Think Tank Certification Program of a large medical technology company that is focused on identifying future health opportunities. We conducted before and after surveys and concluded that the proposed initiatives were impactful in developing an innovative design thinking approach. Participants' awareness and enthusiasm were raised, including their willingness to implement taught skills, values, and methods in their working projects. We conclude that a new curriculum based on HTID is essential and needed to move the needle of healthcare activities from treating sickness to maintaining health.

## Introduction

Innovation has been defined as the result of implementing new or improved products/services/processes to enhance a specific value (1). In healthcare, innovation represents a novel technology, service, or care process that aims to bring benefits compared with previous methods due to its usability and desirability (2). Although an urgent need to facilitate the healthcare system while moving the value from diseases and treatments to patient care and prevention, innovation results faster and wiser in other sectors than in healthcare.

Nowadays, several issues should be addressed to face challenges when implementing innovation in the health domain: needs for funding, use of advanced technologies, a patient-centric approach, the possible need and adoption of a new health business model, payments processing and invoicing, cyber- and data security, regulatory changes and approvals, increasing costs of healthcare delivery, investment in IT procedures and many others (3). Moreover, the focus on short-term (3–5 years) rather than long-term (>10 years) developments has solely the effect of generating inaccurate forecasting on the future of healthcare while preventing innovation from being disruptive (4).

How can we imagine healthcare in 10 years? What will be the effects of available tools and devices for prevention and prediction on diagnosing and treating diseases and on a healthy longevity? How do we deal with inequalities in healthcare delivery, access and increasing costs? Is the current education geared toward the anticipated changes? Questions need answers, and the proper problem identification leads to innovative, applicable solutions.

The current way of training and educating future clinicians, biomedical engineers, health IT, and artificial intelligence (AI) experts in education silos does not lead to disruptions but rather to incremental innovations (5–10). The necessity for innovative and adaptive approaches to improve outcomes brought us to think about a health innovation related adoption of the Design Thinking Approach; a novel way of problem-solving that aims to find the best fit-solution between the customer profile and a new product/service/process, quickly prototyped, and iteratively refined (11).

When compared to traditional problem-solving methods, design thinking brings sustainable and applicable solutions, facilitating improvements for patients, care facilities and communities, while improving management and collaborations toward public health procedures. Based on the outcomes introduced by this approach a closer look at the traditional educational curricula in and around health-related programs (engineering, natural science, clinical science), currently lacking twenty first-century skills (e.g., problem-solving, stakeholder empathy, curiosity stimulation, entrepreneurship, and health economics) is needed. Abookire et al. integrated Design Thinking to develop a workshop through the collaboration between the Health Design Lab and Colleges of Medicine and Population Health at Thomas Jefferson University to enrich traditional public health education curricula (11). The workshop aimed to train public health students to more efficiently and effectively deal with complex problems as future healthcare professionals and providers. Students were engaged to investigate public health problems by applying viable and feasible solutions, demonstrating the valuable role of Design Thinking as an innovative and empathy-driven approach in improving the health of individuals and the wellness of the entire community.

Results from the survey evaluation indicated that the familiarity with design thinking approach procedures increased enormously through the workshop. The students started to realize their abilities to implement meaningfully key concepts of the taught approach. Moreover, students demonstrated a positive attitude toward the event, considering it relevant and applicable in their current academic path and professional career. Participants were also given 10 min to generate low-fidelity prototypes. Ideas included the generation of devices to assist with schedule management and mobile application interfaces to ease physical movements and dietary changes.

On a similar perspective, a Lean Design Thinking approach has been suggested (12), which is an innovative model intended to merge the design thinking and lean startup strategies to help entrepreneurs and intrapreneurs by improving their current projects. The lean design thinking approach can be considered a source of inspiration toward innovation, adopting relevant tools and methods of both strategies, managing and generating business innovation with a customer-centered design. With increased attention toward social and environmental determinants of health (13–15) the study of entrepreneurial-driven public health innovation emerged as one of the ultimate approaches to generate innovative interventions, products, and services by addressing public health issues (14, 16).

Becker et al. (17) investigated the perceptions of graduate public health students regarding Public Health Entrepreneurship (PHE) (17); the application of entrepreneurial skills to accomplish public health missions (18), and their training needs for becoming future health professionals (19, 20). As the first research exploring perspectives of PHE in the academic setting, results from the study demonstrated positive outcomes. PHE was offered to be introduced in the current curriculum where courses incorporated the Council on Public Health Education (CEPH) competencies, actively involving students. Public health trainees were stimulated to apply wealth knowledge into action by combining the existing public health training methods with new social innovation and entrepreneurship (16, 21). Several advantages were highlighted when implementing PHE. Such as the correct identification of evidence-based solutions accompanied with the active ideation and application of prototypes to ameliorate health (22) and the possibility to engage stakeholders involved in public health even beyond sectors and institutions traditionally associated with health. This study confirmed that PHE could be the new way to increase resources by facing twenty first-century challenges in public health across several disciplines or sectors aligned with CEPH competencies. Moreover, the need for specific educational programs in life science technology innovation was previously anticipated by Yock et al. (23) and (9).

Design thinking and entrepreneurship education are considered major drivers behind and to create successful innovation. The Biodesign Program at Stanford University provides a map of needs-driven MedTech innovation processes (identification–invention–implementation). Focused on training and educating students with a specific curriculum that integrates design thinking and commercialization processes, paving the way toward translational medicine (TM). In this context, Foty et al. (24) proposed an innovative curriculum design aimed at teaching scientists and leaders in the field of TM. A new curriculum was created to analyze the business scientific and regulatory aspects of TM, explore the challenges encountered by health professionals, develop critical thinking and communication skills by introducing the topic to a wide range of learners. TM is a new field of study that focuses mostly on integrating an idea, advancing clinical testing, and the final development of new technologies or drugs. For this reason, a broad set of skills are required and included in the TM program. Besides core concepts (e.g., ethics, regulations, funding, policy, etc.), TM skills include effective communication, interdisciplinary, personal reflection, and interprofessional collaboration.

**Figure 1:**
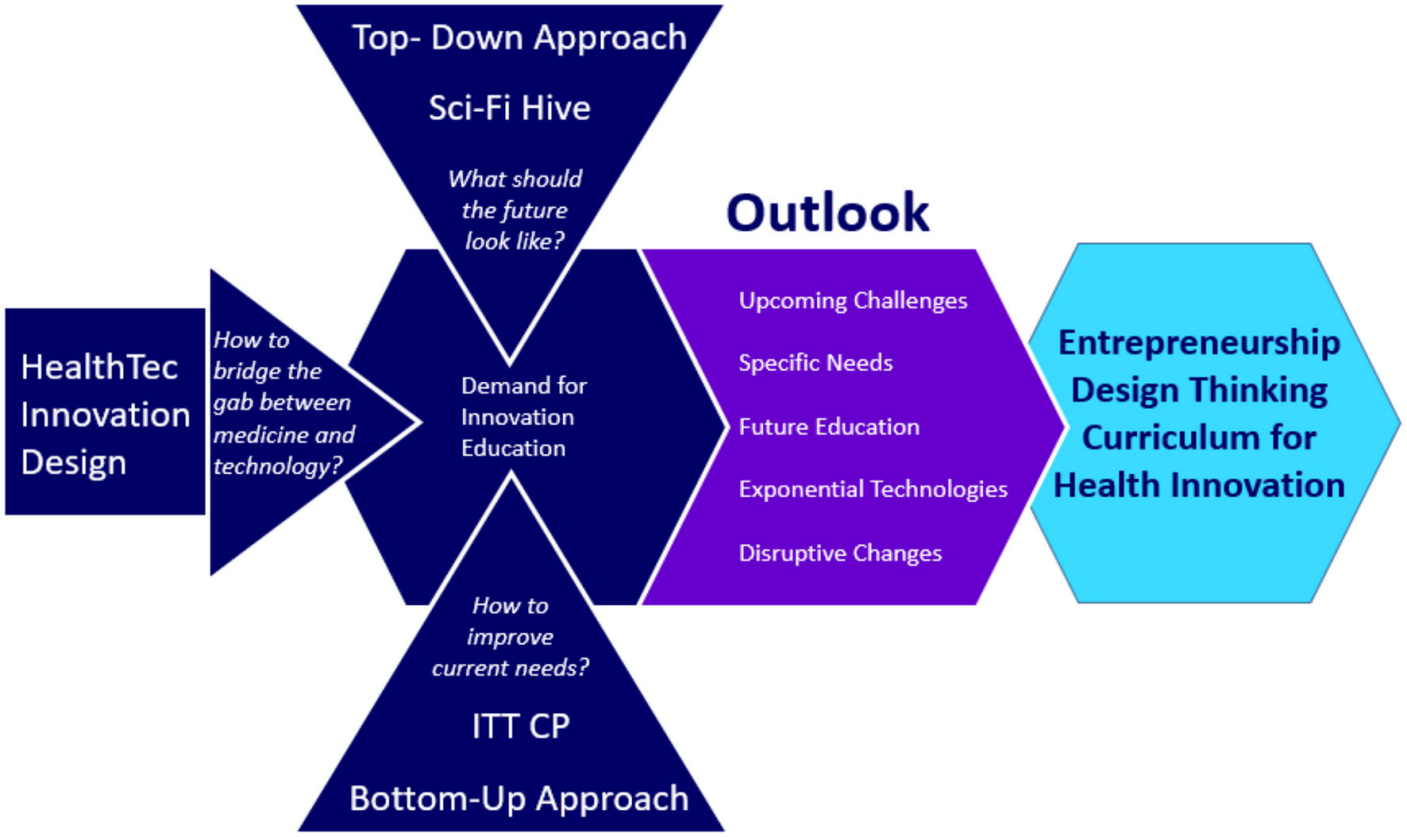
Approaches for a new curriculum.

Although the abundance of ideas and research projects in implementing a design thinking approach raises innovation in a health curriculum, these methods are not widely taught. The present research will describe a series of educational activities to advance health tech innovation. We developed a novel lecture titled HealthTec Innovation Design (HTID) offered for medical students and biomedical engineers that teaches a futuristic view and application of exponential trends. Besides that, we implemented a novel approach using the Purpose Launchpad meta-methodology combined with other innovative tools to define and further exploit an actual project. Additionally, we initiated and promoted with global teams the Sci-Fi Hive Future of Health, a science fiction comic creation event looking 20 years into the future; and the Innovation Think Tank Certification Program (ITTCP, by Siemens Healthineers) focused on the future of health, based on medical technologies with a mid-term vision of a large Medical Technology company. Surveys were conducted to investigate the effectiveness of these events in stimulating and enhancing awareness, curiosity, and expertise toward applying advanced design thinking methods in the field of health tech innovation. The presented research study aims to create the base for establishing a new educational curriculum in Health Technology Innovation Design by integrating advanced methods to prepare future healthcare professionals leading to disruptive and exponential innovation (see Figure 1).

## Materials and Methods

For the understanding and process description in complex systems, such as the healthcare sector, the principles of top-down and bottom-up design were used:

Top-down, based on a global view, the abstract becomes more specific and increasingly subordinate; an overall problem is divided into sub-problems. For this, the Sci-Fi Hive event provided a vision of a great and whole future that is always more detailed and specially designed and formulated.

Bottom-up in that context means the opposite direction. One starts with a specific problem and concludes with the general and higher-level. The ITTCP was used for that point of view. It started with a clinical problem (i.e., coronary artery disease) and used potential pharmaceutical, technical, and organizational solutions for prevention, prediction, diagnosis, intervention, care, and aftercare.

Thus, two fundamentally different ways to understand, describe and present the complex future health issues were employed. Both are used to recognize the future—and with that, the effect on current—needs to adapt the education for bioengineering-, and medical- students, as well as for related fields (e.g., health economics, data sciences, computer sciences). Before and after the events, a qualitative survey was carried out to check relevant characteristics and provide information about them systemically.

### Sci-Fi Hive

Eight teams of 11 participants were put together and assigned to different healthcare topics: the democratization of healthcare, future of emergency/care hospitals, future of homecare, future of increased health-span/longevity, future of health diagnostic, and future of overall health/wellness. After a short introduction to the future of healthcare and exponential innovations, the event was conducted into four main stages.

In the first stage, teams met individually to know each other and brainstorm the first ideas on the chosen healthcare topic.

In the second stage, teams started creating comic characters and developing the hero's journey story around the future vision.

In the third stage, before starting with the prototyping, teams described and scripted the hero's journey story into a comic book format.

Finally, the teams were ready to create and prototype the science fiction comic book cover and individual story panels in the last stage.

Each stage was followed by a feedback session in which teams had the opportunity to share their learning and insights. In total, the event lasted 8 h. The realized Sci-Fi Hive comic book is provided here in Friebe et al. (25).

We designed a pre-and post-event survey in English language using GOOGLE Forms consisting of 14 questions pre-, and 13 questions for the post-event survey as multiple choice, checkboxes, three or five-point Likert scale, short answer or yes/no modalities. Thirty-eight responses were collected from the pre-survey analysis, and 29 responses were collected from the post-survey analysis. Pre- and post-event survey questions and answers are listed in Appendix A in Supplementary Material.

### Innovation Think Tank Certification Program

Innovation Think Tank Certification Program (ITTCP) is an “experiential learning training” based on the experience of successful implementation and management of Innovation Think Tank programs and innovation labs at Siemens Healthineers and several prestigious institutions worldwide. During the ITT program, interdisciplinary participants work in teams using the ITT approach to generate strategic content that helps Siemens Healthineers shape the technology and disease pathway strategy. Also, it helps the host organizations (customers) define concrete projects for further deep dives and research in the ITT lab. The interactive program is designed to develop creative pioneers capable of delivering innovative and customer-centric solutions to the world's most significant challenges in Healthcare in their field of profession.

For the data collection we designed a pre-and post-event survey in English language using GOOGLE Forms consisting of 14 questions pre-, and 11 questions for the post-event survey, again as multiple choice, checkboxes, five-point Likert scale, short answer or yes/no modalities. Forty responses were collected from the pre-survey analysis, and 28 responses were collected from the post-survey analysis. Pre and post-survey questions and answers are listed in Appendix B in Supplementary Material.

The survey answers from the Sci-Fi Hive and ITTCP were statistically analyzed based on the frequency distributions. The frequencies were computed based on the median distribution. In particular, the most frequent answers were transformed into their valid percentage.

### HealthTec Innovation Design Lecture

At the Otto-von-Guericke-University (OVGU) in Magdeburg, Germany, we developed a semester-long lecture titled HealthTec Innovation Design (HTID) offered for medical students and biomedical engineers that teaches a futuristic view and application of exponential trends (23). The HTID, rated 5 ECTS, consists of 10 online lectures with 35 academic hours of teaching, and an additional 90 h of personal and team project assignments. Examples of personal assignments were identifying the personal Massive Transformative Purpose (MTP) and writing a manuscript in a research article format. Students were also asked to forecast and design the future of their current research/education project. Two interdisciplinary teams were formed during the lectures to exploit an actual project leading to developing health-tech innovative ideas. The teams were asked to develop the project using the Purpose Launchpad meta-methodology tool, the OpenExo tools, the classical Business Model Canvas and the Stanford Biodesign approach. In addition, teams were asked to write a short manuscript dedicated to their project proposal in a research article format. A final online examination with a multiple-choice test and a team project presentation concluded the semester earning a certificate of attendance of passed examination.

In further detail, during the lectures, a novel conceptual tool of identifying, validating, and implementation innovation using the Purpose Launchpad (26) was adapted to the healthcare field and combined with other innovative OpenExo tools (27), such as the OpenExo Canvas, to define and further exploit an actual project.

**Figure 2:**
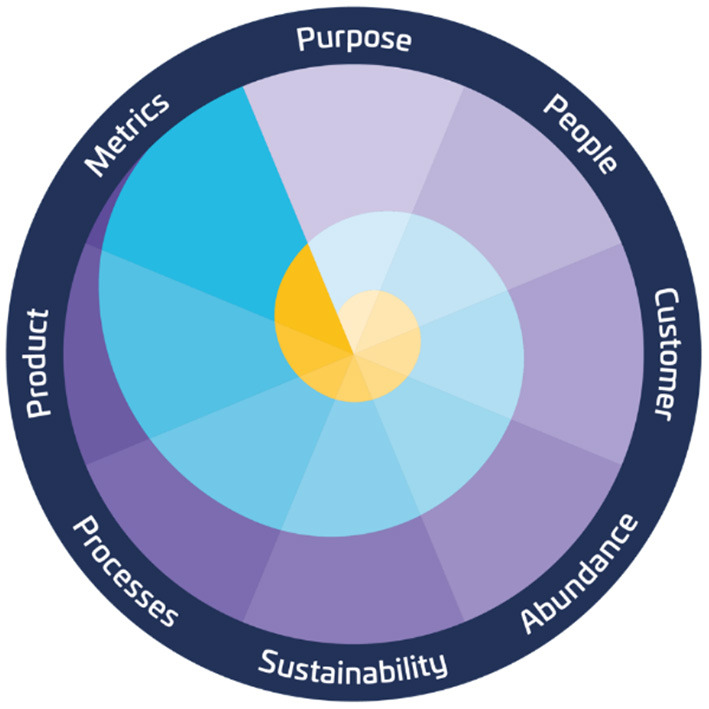
Purpose Launchpad meta-methodology. The eight principles around which the Purpose Launchpad meta-methodology is articulated are represented: purpose, people, customer, abundance, sustainability, strategies, product, and metrics.

The Purpose Launchpad is a meta-methodology to evolve early-stages ideas into purpose-driven, exponential organizations generating massive impact. The Purpose Launchpad is defined as a mindset, a framework, and a methodology to develop an adequate innovative organization, business, product, or service. Moreover, this meta-methodology is articulated around eight principles (see Figure 2) purpose over a problem and problem over a solution, exploration over-optimization, talking to customers over market research, abundance over scarcity, sustainability over investment, mindset over processes and tools, validated learning over product building, qualitative over quantitative metrics.

**Figure 3:**
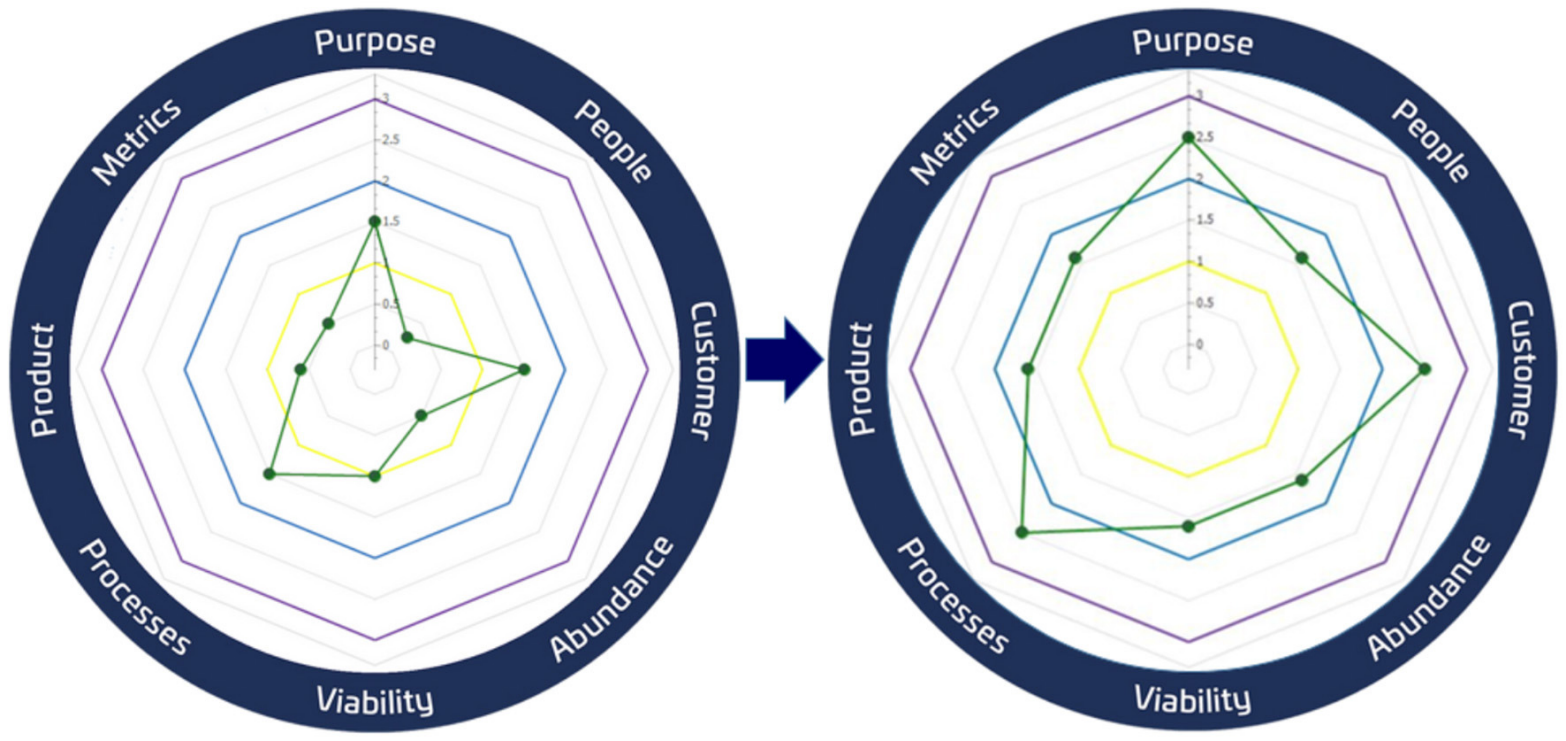
Purpose Launchpad meta-methodology. To enhance learning, the Purpose Launchpad evaluates progress through constant assessments. In this way, the team makes real progress evolving the Purpose Launchpad eight principles over daily/weekly meetings.

The Purpose Launchpad can be applied as a set of principles or an iterative process that evolves continuously over the above-mentioned key area axes (purpose, people, customer, abundance, sustainability, strategies, product, and metrics). To enhance learning, the Purpose Launchpad includes evaluating progress through constant assessments over three evaluation levels: discovery, validation, and growth. Lastly, through innovation Sprints, the team makes real progress evolving the Purpose Launchpad Axes over daily/weekly meetings (see Figure 3).

## Results

### Sci-Fi Hive

Most attendees were male entrepreneurs (35–50 years old) interested in exploring innovative healthcare (86.8%) and mainly new to similar events from the pre-survey analysis. When asked which innovative technology is already implemented (3–5 years' perspective) in participant's work/project, digital healthcare resulted in the most common response (68.4%). Differently, when speculating about the future implementation of technologies (>10 years' perspective), AI (65.8%), VR/AR (57.9%), brain-computer interfaces, or digital healthcare (55.3%) resulted in the most selected responses.

Prevention over treatment, patient empowerment, and personalized medicine was considered the most impactful values/perspectives to generate meaningful innovation in healthcare. Similarly, competencies (problem-solving, collaboration, creativity, communication) and character qualities (curiosity, persistence, adaptability, leadership, initiative, social awareness) were considered very important “innovation mindset” skills over literacy. Moreover, participants defined *innovation* as “the translation of an existing product/service/process into something more efficient/effective/competitive” (31.6%). They reported that the most relevant reason for failure in a startup/business/research/industry project dealing with healthcare innovation is designing a product without considering the customer profile/market test (36.8%). Lastly, several factors were identified as responsible for the prevention of disruptive innovation, such as regulatory approvals, government/political interests, traditional not transparent business model, and fear of changes, as well as, attendee agreed that the gap between scientific literacy and application is not widely exploited by the current university-based education (73.3%).

Moreover, most responders were male entrepreneurs and medical doctors (>35 years old) from the post-survey. Participants reported that the Sci-Fi Hive highly matched their expectations, finding it very informative and insightful (92.6%). Several terms were collected when we asked to describe the most meaningful Sci-Fi Hive take-away in one word. The most common words were *collaboration, creativity, teamwork, insightful, engaging, enlightening, excited, fiction, comic, innovation, imagination, inspiring, relaxed, interaction, fun, big vision, discussion, diversity, interesting, members, think out the box, and great*.

Furthermore, participants declared to be willing to implement the learnings from Sci-Fi Hive into their work/life to improve a current research/business/education project (65.5%) and that the taught methods were likely to raise innovation in their current projects. We asked which growth mindset perspective Sci-Fi Hive has stimulated. Based on a growth mindset approach, participants mainly reported that they were more willing to “try new things” (69%), that “challenges help me to grow” (51.7%). Moreover, “optimistic thinking,” “passion and purpose,” and “long-term thinking” were the most relevant mindset/thinking strategies to raise innovation. In conclusion, participants reported that Sci-Fi Hive was very impactful in stimulating their awareness toward the challenges behind healthcare innovation and that twenty first-century skills in problem-solving, critical thinking, creativity, communication, and collaboration are fundamental to grow an innovative mindset.

### Innovation Think Tank Program

From the pre-survey analysis, most attendees were female students (18–24 years old) interested in exploring innovative healthcare (65%) and mainly new to similar events. When asked which innovative technology is already implemented (3–5 years' perspective) in participant's work/project, digital healthcare resulted in the most common response (47.5%). Differently, when speculating about the future implementation of technologies (>10 years' perspective), AI (57.5%), digital healthcare (55%), and VR/AR (50%) resulted in the most selected responses. Moreover, when asked which factor comes into mind when thinking about healthcare, the top 3 answers were “medical devices and technologies” (77.5%), “healthcare management” (42.5%) and “diseases” (40%), and that “treatment over prevention” has been classified as the main problem in the current healthcare delivery (47.5%). Prevention over treatment, personalized medicine, digital health procedures, and patient-centric approach was considered very impactful values/perspectives to generate meaningful innovation in healthcare. Participants defined innovation as “the translation of an existing product/service/process into something more efficient/effective/competitive” (35%). Lastly, several factors were identified as responsible for the prevention of disruptive innovation such as government/political interests, regulatory approval (e.g., CE, FDA), no transparent business model/markets, traditional/rigid education system, and a long time in the process of implementing new technologies, as well as, participants identified “training of twenty first-century skills” (45%) the main factor to close the gap between scientific literacy and feasible application to improve healthcare. Moreover, most respondents were female students (25–34 years old) from the post-survey. Participants reported that the ITTCP matched their expectations, finding it very informative and insightful (89.3%). Several terms have been collected when asked to describe the most meaningful ITTCP take-away in one word. The most common words were holistic view, mandate, teamwork, vision, interdisciplinary, informative, problem identification, methodology, insightful, enlighten, inclusivity and structure. Furthermore, participants declared to be willing to implement the learnings from ITTCP into their work/life to improve a current research/business/education project (60.7%). “Customer-centric thinking,” “rapid experimentation,” and “passion and purpose” were the three most crucial mindset/thinking strategies to raise innovation. In conclusion, participants reported that a “deep understanding of the problem to be solved” (46.4%) is the most challenging factor when implementing an innovation strategy/methodology to commercialize an invention. That “empathic and collaborative networks” (35.7%) is the most crucial factor needed to switch from the current healthcare methods to innovative healthcare strategy-approach and that “training of twenty first-century skills” the main factor to close the gap between scientific literacy and feasible application to improve healthcare (46.4%).

To summarize, the results obtained from Sci-Fi Hive and ITTCP can be compared, although some questions we provided were different between the two programs. In general, with a top-down approach, Sci-Fi Hive identified prevention over treatment, patient empowerment, and personalized medicine as the most impactful values/perspectives to generate innovation in healthcare, and that regulatory approvals, government/political interests, traditional not transparent business model, fear of changes were responsible factors to prevent disruptive innovation.

When forecasting the future implementation of technologies (>10 years' perspective) to generate innovation in healthcare, attendees reported that digital healthcare, AI, VR/AR and brain-computer interfaces would be the most preferred technologies. Moreover, twenty first-century skills were recognized as fundamental to grow an innovative mindset. Similarly, with a bottom-up approach, ITTCP identified the same factors as the most impactful values to raise innovation and those factors that prevent disruptive innovation and those technologies that preferably would be implemented in >10 years' perspective. Twenty first-century skills were again identified as necessary competencies needed to close the gap between scientific literacy and feasible application in healthcare. Attendees were satisfied and willing to implement the learnings from both events to improve their current research/educational project. A considerable difference between Sci-Fi Hive and ITTCP is related to the type of audience participating in these two programs, and so the way they would apply the learning into their current work project. From Sci-Fi Hive, most attendees were male entrepreneurs (35–50 years old), whereas from the ITTCP attendees were mainly female students (18–24 years old), both categories interested in exploring innovative healthcare and mainly new to similar events. This factor results relevant when considering the educational/business meaning and goals behind these two events. Indeed, ITTCP starting with a bottom-up approach aims to identify clinical needs and search for possible solutions to generate a high-level frame of solutions. This approach would have a meaningful impact when educating and training future healthcare professionals because it teaches methods and strategies to solve unmet clinical needs. On the other side, Sci-Fi Hive starting with a top-down approach, aims to create a great and futuristic vision that can be successively divided into its parts, to make it happen. In this case, the approach can be relevant for healthcare entrepreneurs interested in translating an existing product/service/process into something more efficient/effective/competitive to solve unmet clinical needs through the best customer/market fit.

### HealthTec Innovation Design Lecture

The semester-long lecture was completed successfully by all eight students from Medicine, Neuroscience, Biomedical Engineering, and Computer Science. The scope of personal assignment contents has been reached by students who have taken out meaningful insights. Divided into two equal interdisciplinary teams, students developed two projects to generate innovative solutions to satisfy unmet clinical needs. Teams demonstrated significant interest and involvement in their project, showing constant learnings during the semester. Moreover, the Purpose Launchpad meta-methodology, the Stanford Biodesign approach and the OpenExo tools were implemented successfully and appropriately. When asked students to present their team projects, presentations satisfied all the requirements, and the final examination was passed with good scores, meaning that students acquired the taught material with passion and purpose. Moreover, the HTID course with interdisciplinary students gave attendees the chance to know each other and exchange their expertise, learning and experiences, an optimal requirement in the perspective of healthcare innovation, and a revised educational curriculum. Finally, positive feedback from students suggested the continuation of this series of lectures with the vision of developing a novel curriculum in health-technology innovation design.

Currently, international differences in the education of the health science industry, the lack of emphasis on global healthcare care needs and interdisciplinary collaboration between healthcare providers, clinicians, research institutes and industries leads to the difficulty of identifying and satisfying clinical needs.

Thus, we aim to develop a novel educational curriculum based on the *I3-EME* as an educational concept (*Identify-Invent-Implement)* (28). The educational and teaching focus would be based on an interdisciplinary approach in which medical and engineering students would merge, working together on advanced clinical solutions based on the taught I3-EME Concept. The I3-EME aims to identify unmet clinical needs, invent feasible solutions and successfully implement them at adequate market needs. New technologies based on AI, AR, 3D, robotics, digital health, ethics, and future societal challenges, in line with medical technologies and services, will change the focus from inpatient to outpatient, prevention, reduction of costs, and democratization healthcare. Based on this educational content and the I3-EME concepts, students will have the opportunity to work and explore meaningful and valuable products/services to understand and solve global healthcare needs.

We proposed a study plan for this novel educational curriculum based on economy/business, medical/clinical/healthcare innovation and engineering study subjects, with corresponding credit points (CP). The medical and the engineering departments would interact with the hospital structures. The study plan will be structured into four semesters in which the subjects mentioned above will be covered (see Table 1).

**Table 1:** Proposed study plan suggestion for a novel master curriculum in Health Tech Innovation Design.

**1. Semester**	**2. Semester**	**3. Semester**	**4 Semester**
Marketing for Healthcare **5CP**	Value Based Technology and Innovation Management **5CP**	Entrepreneurial Finance and Venture Capital **5CP**	Master Thesis **20CP**
Market Research and Business Modeling **5CP**	Medical Innovation Needs 1 – Clinical Input (MI1) **5CP**	Discover UNMET CLINICAL NEEDS in a clinical Innovation Lab – think Entrepreneurial (UCN) **10CP**	
Innovation to Healthcare Democratization **5CP**	Individual Healthcare International – application of Value Proposition Canvas and Biodesign Principles (IHI) **5CP**		Health Economics and Reimbursement **5CP**
Healthcare Technology Innovation – future developments with high impact and need for change (HTI) **5CP**	Exponential Technologies and Designs for Extreme Affordability – Healthcare related (EXP) **5CP**	Medical Innovation Needs 2 – Screening, Diagnosis, Therapy, Prevention, Inpatient vs. Outpatient (MI2) **5CP**	Healthcare related Regulatory issues + Medical Product Risk Analysis (REG) **5CP**
20CP	20CP	20CP	30CP

*The study plan articulates into four semesters covering economic/business with 50 CP (blue color), medical/clinical/healthcare innovation with 50 CP (purple) and the Innovation Lab in cooperation with the hospital with 10 CP (orange color)*.

## Discussion

How can we imagine healthcare in 10 years? What will be the effects of prevention and prediction on diseases and healthy longevity? How do we deal with inequalities and increasing costs? Is the current education geared toward the anticipated changes? We started with these questions to identify a proper solution.

When thinking about healthcare today, several obstacles should be addressed to overcome the current status and raise innovation. The main factors are the ever-increasing cost of healthcare provision, the disparity in quality care among countries and even inside countries from rural to urban, insufficient health insurance coverage, lack of empathy and communication between patient-providers, traditional and unilateral approaches, and fear of implementing new technologies.

These are just a few of the challenges that the healthcare system is facing nowadays. Although the urgent need to innovate and improve the healthcare system and services, the entire setup and management typically only leads to incremental rather than disruptive innovation. Incremental means that we observe improvements that do not significantly impact longevity but increase the cost significantly based on existing technologies and workflows.

One reason for this fact could be the current way of educating and training future clinicians, biomedical engineers, health IT, and AI experts in silos. The lack of transferability of scientific literacy to applicable solutions prevents the transformation of knowledge and ideas into innovative, feasible products to satisfy unmet clinical needs.

To close the gap between scientific literacy and application, we wanted to develop a novel lecture (dubbed HealthTec Innovation Design) for medical students and biomedical engineers that teaches a more futuristic view and includes applying exponential technologies in combination with teaching intentional disruption. We implemented a novel approach using the Purpose Launchpad meta-methodology and the Stanford Biodesign approach to define, experimentally validate and further exploit deep problem understanding to formulate an actual innovation project.

The learning from the global Sci-Fi Hive, a science fiction comic creation event, and the Innovation Think Tank Certification Program, both focused on the future of health but with different starting points, highlighted the need for a novel curriculum approach.

Through the implemented online surveys, we investigated the quality and efficiency of these educational programs and events. The survey results showed that most attendees were entrepreneurs, medical doctors, and students interested in exploring the topic of innovative healthcare. When speculating about the future implementation of technologies (>10 years' perspective), most responses were digital healthcare, AI, VR/AR, and brain-computer interfaces. Concepts like prevention over treatment, patient empowerment, and personalized medicine were considered the most impactful values/perspectives to generate meaningful innovation in healthcare. Factors like regulatory approvals (e.g., CE, FDA), government/political interests, unclear business model, and fear of changes were identified as responsible for preventing disruptive innovation. The lack of a customer profile/market test was the main reason for failure in a start-up/business/research/industry project dealing with healthcare innovation.

Moreover, innovation has been defined as the translation of an existing product/service/process into something more efficient/effective/competitive, and that twenty first-century competencies were considered very important “innovation mind-set” skills leading to innovation. More empathic and collaborative networks were identified with a deep understanding of the problem to be solved, respectively, as challenging and needed factors to generate an innovative healthcare strategy approach. Furthermore, attendees were satisfied regarding the overall programs/events outcome and willing to implement the taught methods to improve their current research/business/educational project. Attendees agreed that the gap between education and research application is still vast, estimating that training twenty first-century skills would be optimal to close this gap. A summary of the learned skills and continents from the different education programs leading to the novel curriculum development is reported in Table 2.

**Table 2:** Summary of the learned skills from the different educational programs—Sci-Fi Hive, ITTCP, HTID—needed to formulate the novel curriculum in Innovation Tech Design.

**Sci-Fi Hive**	** ITT CP**	** HTID**	**Novel Curriculum in Healthtech Innovation Design**
Twenty-First century skills	Twenty-First century skills	Twenty-First century skills	Twenty-First century skills
Interdisciplinary teams	Interdisciplinary teams	Interdisciplinary teams	Interdisciplinary teams
Top- Down Problem solving approach toward Innovation	Bottom- Up problem solving approach toward Innovation	From lectures (literacy) to the development of innovative projects (application)	Academic Transfer Strategies/Commercialization of Research Results (from literature to application)
The hero's journey story	The ITTCP methodology	Purpose Launchpad meta-methodology, OpenExo tools, the Stanford Biodesign approach	The HTID teaching methodology, the Stanford Biodesign approach, I3-EME-Concept
“Try new things,” “challenges help me to grow,” “passion and purpose,” “optimistic thinking”	“Customer-centric thinking,” “rapid experimentation,” “passion and purpose”	“Growth mindset” approach	The HTID teaching methodology, the Stanford Biodesign approach, I3-EME-Concept
Fun, curiosity, creativity, interdisciplinary interaction	Structure, insight, methodology, interdisciplinary interaction	“Thinking out of the box,” “learning from mistakes” approach, creativity, methodology, personalization, empathy, interdisciplinary interaction	International academic and industrial collaboration across countries to identify individual needs of the global healthcare challenges

Based on our research results and the need of a revised education, our mission is to design a novel Master's Degree, called *Entrepreneurship Design Thinking Curriculum for Healthtech Innovation*, based on health technology innovation design, digital health methods, predictive and preventive medicine to reach our transformative goal in democratizing healthcare. Hence, we aim to establish novel curricula combining technical, economic, scientific and medical skills with twenty first-century skills to educate future health innovators and professionals. These curricula would comprehend programs taught in English, online teaching, on-site team projects and annual summer/winter schools. Through individual assignments, trimestral examinations, research team projects and tutoring support, students would be capable of reaching a novel degree in innovation generation aimed to generate the innovative mindset, attitude, and learning skills behind the feasible, valuable application of disruptive health technologies and finally moving the healthcare needle from sickness to health.

The master curriculum for Health Tec innovation design primarily aims at three interface areas for clinical innovation:

Healthcare economics (blue colored): Methods of health economic evaluation (benefit assessment, cost assessment, direct costs, indirect costs) play a significant role concerning healthcare democratization and require a deep understanding of economic processes and reimbursement for medical effectiveness and economic efficiency. Students are trained in a financial analysis perspective and can decide on broad expertise in various economic backgrounds for research and innovation projects.

Innovation Methodologies (purple colored): with various agile innovation methods in product development, students can resolve any problem quickly and in a goal-oriented manner. In addition to the basics and the constant exchange in interdisciplinary groups, the students also learn to apply the methods they have learned in real projects.

Application-driven research (orange color): Students cooperate with the Innovation Lab and clinical departments to apply economic knowledge and innovation methodologies to detect unmet clinical needs, solve them with the newest approaches, and change the whole process.

## Conclusion

Currently, university-based educational programs lack twenty first-century skills and innovative approaches, essential for identifying and implementing exponential technologies designed to cover unmet clinical needs. The nowadays trend is to look at innovation as just an incremental process, disregarding what is instead disruptive. To overcome these limits and stimulate innovative thinking, we developed a new lecture titled *HealthTec Innovation Design* for clinical and biomedical engineering students to teach a novel methodological approach to develop and implement disruptive health technologies.

Moreover, *Sci-Fi Hive*, a science fiction comic event, and *Innovation Think Tank Certification Program* raised interest and awareness toward a growth mindset behind disruptive innovation. From the survey results, we can conclude that our educational and initiative programs have impacted a growing interest in innovation, focusing on a distinctive design thinking approach. Participants raised awareness toward those values and perspectives needed to overturn the innovation process from incremental to disruptive, from literacy to valuable competencies and feasible applications. The programs developed the basement of a creative growth mindset, sharing tools and methods necessary when identifying and implementing a new product/process to detect and fulfill unmet clinical needs.

Moreover, participants reported being enthusiastic and willing to implement these new skill sets and methods to enhance their current research/business/educational project solicited by passion, purpose, and optimistic thinking.

Prevention, prediction, personalization, empathy and democratization; with different skills and innovative setups, we can design the future of health toward exponential medicine. Based on our results, we are convinced that developing a new curriculum based on HTID and educational programs/events such as Sci-Fi Hive and ITTCP would be essential. Hence, our vision is to raise the awareness needed to upgrade the global way of training and educating healthcare professionals enhancing the future of healthcare.

## Data Availability Statement

The raw data supporting the conclusions of this article will be made available by the authors, without undue reservation.

## Ethics Statement

Ethical review and approval was not required for the study on human participants in accordance with the local legislation and institutional requirements. Written informed consent for participation was not required for this study in accordance with the national legislation and the institutional requirements.

## Author Contributions

BB and HF carried out the experiment, wrote the manuscript with support from MF, and designed the surveys. MM and SH supported us with respect to Siemens Healthineers Innovation Think Tank Certification Program. MF conceived the original idea and supervised the project. All authors contributed to the article and approved the submitted version.

## Conflict of Interest

MM and SH are employed by Siemens Healthineers, Erlangen, Germany. The remaining authors declare that the research was conducted in the absence of any commercial or financial relationships that could be construed as a potential conflict of interest.

## Publisher's Note

All claims expressed in this article are solely those of the authors and do not necessarily represent those of their affiliated organizations, or those of the publisher, the editors and the reviewers. Any product that may be evaluated in this article, or claim that may be made by its manufacturer, is not guaranteed or endorsed by the publisher.
