# Supplementary material for: A Structured Pathway Toward Disruption: A Novel HealthTec Innovation Design Curriculum With Entrepreneurship in Mind
Source: Front Public Health. 2021 Sep 3;9:715768. doi: 10.3389/fpubh.2021.715768 (PMC8446366; doi:10.3389/fpubh.2021.715768)
Supplement: Supplementary file 1 [file Table_1.docx]

Supplementary Material

# Supplementary Figures and Tables

The pre and post surveys are recorded in the following supplementary files.

**Appendix A**

| **Pre Sci-Fi Hive Survey - Questions** |
| --- |
| 1.     Age  2.     Sex  3.     Educational / Job Background  4.     What is your goal / expectation by attending Sci-Fi Hive?  5.  Did you already participate in events / educational programs leading to healthcare innovation?  6.     If yes, which one?  7.     Which Innovative Technology are you already implementing to improve the future of healthcare (i.e., 3-5 years’ approach)?  8.     Which technology would you imagine to implement in 10 years to improve the future of healthcare?  9.     Which “Value / Perspective” should be the most impactful to generate meaningful innovation in the healthcare system? Rate your answer from 1-3; where 1 = less impactful, 2 = impactful, 3 = very impactful  10.  Which skills would be needed to stimulate an „Innovative Mindset"? Rate your answer from 1-3; where 1 = less important, 2 = important, 3 = very important  11.  How would you define “Innovation”?  12.  Which is the most important reason for failure in most innovative healthcare Startups / Industries / Research-Business projects?  13.  Which factors do you believe prevent “Disruptive Innovation"? NB: Disruptive Innovation is the process of changing a workflow/product completely, dramatically reducing the cost/time/access; Rate your answer from 1-3; where 1 = less important, 2 = important, 3 = very important  14.  Do you think basic university education closes the gap between scientific literacy and feasible application to improve healthcare (i.e., Patient’s needs)? |

| **Pre Sci-Fi Hive Survey - Answers** |
| --- |
| ●      18-24  ●      25-34  ●      35-50  ●      >50 |
| ●      Male  ●      Female  ●      Diverse |
| ●      Medical Doctor  ●      Entrepreneur  ●      Employee  ●      Research/Scientist  ●      Bioengineer  ●      Business Advisor  ●      Student |
| ●      Exploring the topic of Innovative Healthcare  ●      Networking  ●      Improving a current Research/Industry project  ●      Fun  ●      Other |
| ●      Yes  ●      No |
| Short-answer text |
| ●      Digital Healthcare  ●      Virtual Reality/Augmented Reality  ●      Artificial Intelligence  ●      3D Printing  ●      Robotics  ●      Brain-Computer Interfaces  ●      Health Wearables  ●      Voice Assistant  ●      New Touch Interfaces  ●      Minimal Invasive Therapy Systems  ●      Environmental Protection and Sustainability  ●      Data Management/Cybersecurity  ●      Electronic and Sensors  ●      Other |
| ●      Digital Healthcare  ●      Virtual Reality/Augmented Reality  ●      Artificial Intelligence  ●      3D Printing  ●      Robotics  ●      Brain-Computer Interfaces  ●      Health Wearables  ●      Voice Assistant  ●      New Touch Interfaces  ●      Minimal Invasive Therapy Systems  ●      Environmental Protection and Sustainability  ●      Data Management/Cybersecurity  ●      Electronic and Sensors  ●      Other |
| ●      Prevention over treatment  ●      Personalized medicine  ●      Patient-centric approach  ●      Value (reimbursement) based outcome  ●      Digital health procedures  ●      Treatment based on high technology systems  ●      Focus on significant cost reduction |
| ●      Foundational Literacies (literacy, numeracy, scientific, ICT, financial, cultural and civic literacy)  ●      Competencies (critical thinking/problem solving, creativity, communication, collaboration)  ●      Character quality (curiosity, initiative, perseverance/grit, adaptability, leadership, social and cultural awareness) |
| ●      A life-changing idea  ●      The development of a new product/service/process  ● The translation of an existing product/service/process into something more efficient/effective/competitive  ●      A very efficient/technologic but expensive product/service/process  ●      Invention x Commercialization |
| ●      Poor/inefficient business model  ●      Not enough funds  ●      Bureaucracy (e.g., regulations, certification, ethics, patents)  ●      Designing a product without a customer profile (i.e., market test)  ●      High competition  ●      Missing network partners |
| ●      Not enough focus on cost reduction  ●      Not enough innovative ideas  ●      Long time in the process of implementing new technologies  ●      Government/politics interests  ●      Traditional/rigid education system  ●      Not clear business model/markets  ●      Fear of changes (e.g. technologies over human control, payment, jobs etc.)  ●      Regulatory approval (e.g., CE, FDA) |
| ●      Yes  ●      No |

| **Post Sci-Fi Hive Survey - Questions** |
| --- |
| 1.     Age  2.     Sex  3.     Educational / Job background  4.     Did the Event match your expectations?  Rate your answer 1-5; where 1 = very unlikely, 3 = neutral, 5 = very likely  5.     Was the workshop informative for you and shared new insights?  6.     Describe in one word the most meaningful SciFi Hive "take away".  7.     How do you plan to implement learnings from SciFi Hive workshop into your current work / life?  8.     How likely is that you will implement the methods suggested to raise innovation? Rate your answer 1-5; where 1 = very unlikely, 3 = neutral, 5 = very likely  9.     Which “Growth Mindset” perspectives did SciFi Hive stimulate in you?  10.  Which "Mindset / Thinking Strategy" is more relevant to you to stimulate innovation? Rate your answer from 1-3; where 1 = less relevant, 2 = relevant, 3 = most relevant  11.  How impactful was the SciFi Hive to stimulate your awareness toward the challenges behind innovative healthcare? Rate your answer 1-5; where 1 = not at all impactful, 3 = neutral, 5 = very impactful  12.  Which skills would be needed to stimulate an „Innovative Mindset"? Rate your answer from 1-3; where 1 = less important, 2 = important, 3 = very important  13.  Do you think basic university education closes the gap between scientific literacy and feasible application to improve healthcare (i.e., Patient’s needs)? |

| **Post Sci-Fi Hive Survey - Answers** |
| --- |
| ●      18-24  ●      25-34  ●      35-50  ●      >50 |
| ●      Male  ●      Female  ●      Diverse |
| ●      Medical Doctor  ●      Entrepreneur  ●      Employee  ●      Research/Scientist  ●      Bioengineer  ●      Business Advisor  ●      Student |
| 5-points Likert scale |
| ●      Yes  ●      No |
| Short-answer text |
| ●      Connect with other scientists’/industries partners  ●      Participate more in similar events/projects  ●      Apply the acquired knowledge to improve my current research/business/education project  ●      Nothing  ●      Other |
| 5-points Likert scale |
| ●      Failure is an opportunity to grow  ●      I can learn to do anything I want  ●      Challenges help me to grow  ●      Feedbacks are constructive  ●      I am inspired by the success of others  ●      I like to try new things  ●      My effort and attitude determine my abilities  ●      Other |
| ●      Passion and purpose  ●      Rapid experimentation  ●      Customer-centric thinking  ●      Risk it, and mitigate it  ●      Long-term thinking  ●      Optimistic thinking |
| 5-points Likert scale |
| ●      Foundational Literacies (literacy, numeracy, scientific, ICT, financial, cultural and civic literacy)  ●      Competencies (critical thinking/problem solving, creativity, communication, collaboration)  ●    Character quality (curiosity, initiative, perseverance/grit, adaptability, leadership, social and cultural awareness) |
| ●      Yes  ●      No |

**Appendix B**

| **Pre Innovation Think Tank Certification Program Survey - Questions** |
| --- |
| 1.     Age  2.     Sex  3.     Educational / Job background  4.     What is your goal / expectation by attending the Innovation Think Tank Certification Program?  5.     Did you already participate in events / educational programs leading to healthcare innovation?  6.     If yes, which one?  7.     Which Innovative Technology are you already implementing to improve the future of healthcare (i.e., 3-5 years’ approach)?  8.     Which technology would you imagine to implement in 10 years to improve the future of healthcare?  9.     What is the first thing that comes to mind when talking about Healthcare? Select the most 3 important options  10.  What do you think is the main problem with current healthcare delivery?  11.  Please rate the most impactful “Value / Perspective” to generate meaningful innovation in the healthcare system. Rate your answer from 1-3; where 1 = less impactful, 2 = impactful, 3 = very impactful  12.  How would you define “Innovation”?  13.  Which factors do you believe prevent “Disruptive Innovation"? NB: Disruptive Innovation is the process of changing a workflow/product completely, dramatically reducing the cost/time/access. Rate your answer from 1-3; where 1 = less important, 2 = important, 3 = very important  14.  Which factor would close the gap between scientific literacy and feasible application to improve healthcare (i.e., Patient’s needs)? |

| **Pre Innovation Think Tank Certification Program Survey - Answers** |
| --- |
| ●      18-24  ●      25-34  ●      35-50  ●      >50 |
| ●      Male  ●      Female  ●      Diverse |
| ●      Medical Doctor  ●      Entrepreneur  ●      Employee  ●      Research/Scientist  ●      Bioengineer  ●      Business Advisor  ●      Student |
| ●      Exploring the topic of Innovative Healthcare  ●      Networking  ●      Improving a current Research/Industry project  ●      Fun  ●      Other |
| ●      Yes  ●      No |
| Short-answer text |
| ●      Digital Healthcare  ●      Virtual Reality/Augmented Reality  ●      Artificial Intelligence  ●      3D Printing  ●      Robotics  ●      Brain-Computer Interfaces  ●      Health Wearables  ●      Voice Assistant  ●      New Touch Interfaces  ●      Minimal Invasive Therapy Systems  ●      Environmental Protection and Sustainability  ●      Data Management/Cybersecurity  ●      Electronic and Sensors  ●      Other |
| ●      Digital Healthcare  ●      Virtual Reality/Augmented Reality  ●      Artificial Intelligence  ●      3D Printing  ●      Robotics  ●      Brain-Computer Interfaces  ●      Health Wearables  ●      Voice Assistant  ●      New Touch Interfaces  ●      Minimal Invasive Therapy Systems  ●      Environmental Protection and Sustainability  ●      Data Management/Cybersecurity  ●      Electronic and Sensors  ●      Other |
| ●      Human Body Structure  ●      Lifestyle  ●      Diseases  ●      Healthcare management  ●      Mental challenges  ●      Healthcare education  ●      Ethic and regulations  ●      Medical devices and technologies  ●      Medical procedures and therapies |
| ●      Sickness over health system  ●      Treatments over prevention  ●      Reimbursement (value) based on procedures over outcomes  ●      One-size fit medicine approach over personalized medicine  ●      Invasive over minimal/ noninvasive therapies  ●      Provider-centric over patient-centric approach |
| ●      Prevention over treatment  ●      Personalized medicine  ●      Patient-centric approach  ●      Value (reimbursement) based outcome  ●      Digital health procedures  ●      Treatment based on high technology systems  ●      Focus on significant cost reduction |
| ●      A life-changing idea  ●      The development of a new product/service/process  ●      The translation of an existing product/service/process into something more efficient/effective/competitive  ●      A very efficient/technologic but expensive product/service/process  ●      Invention x Commercialization |
| ●      Not enough focus on cost reduction  ●      Not enough innovative ideas  ●      Long time in the process of implementing new technologies  ●      Government/politics interests  ●      Traditional/rigid education system  ●      Not clear business model/markets  ●      Fear of changes (e.g. technologies over human control, payment, jobs etc.)  ●      Regulatory approval (e.g., CE, FDA) |
| ●      Training of 21st century skills (critical thinking, creativity, collaboration, communication)  ●      Empathy towards the user (patient, medical doctor)  ●      Specialized training for interdisciplinary interface managers  ●      Academic transfer strategies/commercialization of research results |

| **Post Innovation Think Tank Certification Program Survey - Questions** |
| --- |
| 1.     Age  2.     Sex  3.     Educational / Job background  4.     Did the event match your expectations? Rate your answer 1-5; where 1 = very unlikely, 3 = neutral, 5 = very likely  5.     Was the Innovation Think Tank Certification Program informative for you and shared new insights?  6.     Describe in one word the most meaningful Innovation Think Tank Certification Program "take away".  7.     How do you plan to implement learnings from the Innovation Think Tank Certification Program into your current work / life?  8.     Which "Mindset / Thinking Strategy" is more relevant to you to stimulate innovation? Select the most 3 important options  9.     Which is the most challenging factor when implementing an innovation strategy/methodology to commercialize an invention?  10.  Which is the most important factor needed to switch from the current healthcare methods to innovative healthcare “strategy-approach”?  11.  Which factor would close the gap between scientific literacy and feasible application to improve healthcare (i.e., Patient’s needs)? |

| **Post Innovation Think Tank Certification Program Survey - Answers** |
| --- |
| ●      18-24  ●      25-34  ●      35-50  ●      >50 |
| ●      Male  ●      Female  ●      Diverse |
| ●      Medical Doctor  ●      Entrepreneur  ●      Employee  ●      Research/Scientist  ●      Bioengineer  ●      Business Advisor  ●      Student |
| 5-points Likert scale |
| ●      Yes  ●      No |
| Short-answer text |
| ●      Connect with other scientists’/industries partners  ●      Participate more in similar events/projects  ●      Apply the acquired knowledge to improve my current research/business/education project  ●      Nothing  ●      Other |
| ●      Passion and purpose  ●      Rapid experimentation  ●      Customer-centric thinking  ●      Risk it, and mitigate it  ●      Long-term thinking  ●      Optimistic thinking |
| ●      Find the best fit-solution for a customer profile  ●      Match different fields of research  ●      Costs of generating a low fidelity prototype  ●      Patenting, funds and bureaucracy  ●      Deep understanding of the problem to be solved |
| ●      Rethinking healthcare delivery  ●      Innovation management in student education  ●      Emphatic and collaborative networks  ●      Exponential technologies |
| ●      21st century skills (critical thinking, creativity, collaboration, communication)  ●      Need-led innovation  ●      Entrepreneurship and Start-Up generation  ●      Incremental development  ●      Subject-specific expertise |
